# Canine Leishmaniosis in Italy: Geospatial Trends and Incidence, a Shift in Epidemiological Approach

**DOI:** 10.1111/zph.70078

**Published:** 2026-07-22

**Authors:** Federica Bruno, Germano Castelli, Eugenia Oliveri, Gaetano Oliva, Mariele De Santi, Claudia La Rocca, Ornella Melideo, Marco Ripamonti, Francesco La Russa, Maria Liliana Di Pasquale, Antonella Galante, Roberto Hamel, Giorgio Schiera, Marzio Cometa, Silvia Scibetta, Stefano Reale, Fabrizio Vitale

**Affiliations:** ^1^ WOAH Leishmania Reference Laboratory Istituto Zooprofilattico Sperimentale Della Sicilia Palermo Italy; ^2^ Department of Veterinary Medicine and Animal Production University of Naples “Federico II” Naples Italy; ^3^ Elanco Italia S.P.A. Sesto Fiorentino Italy

**Keywords:** canine leishmaniosis, dogs, epidemiology, incidence, Italy, zoonoses

## Abstract

**Introduction:**

Canine leishmaniosis (CanL) has shown a marked geographic expansion in Italy, progressively affecting central and northern regions previously considered non‐endemic. Laboratory‐based prevalence estimates remain informative in well‐designed surveys; however, prevalence derived from diagnostic submissions may be influenced by study design (e.g., convenience sampling and clinical bias), diagnostic performance and cut‐offs, and incomplete demographic denominators, limiting representativeness and comparability.

**Methods:**

We estimated a provincial incidence proxy for CanL during 2022–2023 by combining newly diagnosed cases from routine diagnostic submissions with canine population approximations derived from ISTAT demographic data, applying a baseline dog‐to‐human ratio of 1:5. Rates were expressed as cases per 10,000 dogs and provinces were classified into five predefined operational risk categories. Robustness was assessed through alternative denominator scenarios (1:4 and 1:6), a restricted registry‐based regional analysis in SINAC‐covered regions, and descriptive comparison with independent epidemiological, entomological, and ecological evidence.

**Results:**

Incidence estimates revealed substantial heterogeneity across Italy, ranging from 0 to nearly 500 cases per 10,000 dogs. Overall, 49 provinces (45.4%) were classified as minimal risk, 30 (27.8%) as low risk, 21 (19.4%) as moderate risk, 4 (3.7%) as high risk, and 4 (3.7%) as very high risk. Most minimal and low‐category provinces were located in northern and central Italy, whereas higher categories were predominantly observed in Southern Italy and Sicily; however, very high provinces were also identified in North‐Western Italy (Liguria), notably Imperia and Savona. While absolute provincial values were sensitive to denominator assumptions, registry‐based analyses in SINAC‐covered regions preserved the regional ranking of incidence, supporting the framework mainly for broad spatial prioritisation rather than precise estimation of absolute burden.

**Conclusions:**

Integrating an incidence proxy alongside prevalence provides a pragmatic surveillance tool for spatial prioritisation and risk communication under real‐world constraints. The framework is most informative for identifying higher‐burden areas and supporting proportionate prevention and control strategies, especially when interpreted together with registry‐based restricted analyses and independent external evidence. Within a One‐Health perspective, incidence‐based indicators in canine surveillance can improve comparability with routinely used human notification metrics and support coordinated planning across sectors, while remaining distinct in epidemiological meaning between hosts.

## Impacts

- This study introduces an incidence proxy (biennial cumulative notification rate) derived from routine diagnostic submissions and an estimated denominator, providing temporally sensitive comparative information to complement prevalence‐based surveillance
- The proposed risk classification enables proportional and context‐specific preventive strategies, supporting One Health surveillance planning.
- Results provide veterinarians and authorities with a practical tool to guide tailored prevention and control actions across different epidemiological contexts.

## 1 Introduction

Canine leishmaniosis (CanL), caused by the protozoan *Leishmania infantum* and transmitted by sand flies of the genus *Phlebotomus*, represents a zoonosis of growing concern in Italy, with implications for both animal health and public health (Alvaro et al. 2025). In Southern Europe, CanL is among the major endemic vector‐borne diseases, characterised by high prevalence and heterogeneous distribution across countries and regions. Italy is one of the most affected, with an average seroprevalence of 14% in highly endemic areas (Morales‐Yuste et al. 2022). In recent years, the infection has progressively expanded into previously non‐endemic areas of Northern Italy, a phenomenon linked to climate change and the confirmed presence of competent vectors at higher latitudes and altitudes than in the past (Dantas‐Torres et al. 2019; Gradoni et al. 2017; Maroli et al. 2013). This epidemiological shift entails an increasing risk of new autochthonous outbreaks and highlights the need for integrated and continuous surveillance of both vectors and canine infection.

Local evidence supports this expansion. A cross‐sectional survey in Parma (Emilia‐Romagna) reported a seroprevalence of 16.8% using IFAT at a cut‐off of 1:80, with a substantial proportion of seropositive dogs born and raised locally (Priolo et al. 2024). These findings suggest active local transmission cycles in previously non‐endemic settings and underscore the importance of region‐specific surveillance that integrates serological, entomological, and climatic data within a One Health framework. More broadly, the Italian epidemiological landscape of CanL has evolved over the past decades, with endemic areas expanding northward as a result of environmental, climatic, and ecological changes, including increased average seasonal temperatures and vector density in newly suitable areas (Gradoni et al. 2022; Maroli et al. 2008; Moirano et al. 2020; Todeschini et al. 2024).

Traditional surveillance has relied on prevalence, that is, the proportion of positive animals in a given population. However, the interpretability of prevalence largely depends on study design (e.g., convenience sampling, inclusion of clinically suspected dogs) and the diagnostic performance of serological tests. Consequently, prevalence estimates can either over‐ or underestimate the true infection burden depending on the sampling frame and test characteristic (Maia and Campino 2008; Solano‐Gallego et al. 2009).

Commonly used serological tests, including IFAT and ELISA, are subject to imperfect specificity and to variability linked to laboratory procedures. IFAT cross‐reactivity with other trypanosomatids has been reported; however, because the circulation of *Trypanosoma* spp. has not been documented in Italy, the epidemiological relevance of this mechanism is likely limited in the national context (Maia and Campino 2008; Mettler et al. 2005). More relevant sources of misclassification include variability in cut‐off titres, differences in antigen preparation and reading criteria, and antibody persistence following exposure (Maia and Campino 2008; Solano‐Gallego et al. 2009). Molecular methods (PCR/qPCR) on tissue samples may improve analytical specificity and, in many contexts, sensitivity; nevertheless, their diagnostic sensitivity can vary according to the sampled tissue and infection stage, and results should therefore be interpreted as complementary to serology in epidemiological studies (Castelli et al. 2021; Coura‐Vital et al. 2013; Solano‐Gallego et al. 2009).

Without correction for diagnostic error, prevalence may misrepresent the true burden of infection. More reliable assessments require sensitive methods, such as PCR on tissue samples, combined with confidence intervals to capture uncertainty (Coura‐Vital et al. 2013; Dujardin et al. 2008). Nevertheless, the absence of longitudinal surveillance systems, the lack of official registries, and the difficulty of monitoring canine cohorts over time remain major obstacles to obtaining robust incidence data. In light of the ongoing expansion of CanL and the detection of new autochthonous foci, several authors stress the need to incorporate incidence studies into regional surveillance through prospective designs and early diagnostic tools (Melo et al. 2018; Moreira et al. 2003; Teixeira‐Neto et al. 2014).

Incidence, defined as the number of new infections occurring over a given time period, provides a more dynamic measure of transmission than prevalence. It is crucial for assessing the current transmission risk in canine populations, monitoring the effectiveness of preventive measures such as repellents or vaccines, and distinguishing autochthonous from imported cases (Paradies et al. 2006). Different approaches (e.g., incidence density rate, annual seroconversion rate) have shown its adaptability to operational contexts and its value for surveillance in endemic areas (Paradies et al. 2006). However, incidence estimates in Italy are hampered by incomplete dog registries: in Rome, only 75.3% of owned dogs were recorded, implying a 25% underestimation (Carvelli et al. 2020). This distortion of the denominator may bias rate estimates and risk classification. To operationalise an incidence‐based surveillance indicator at national scale despite incomplete canine registries, we combined newly diagnosed cases with a proxy denominator derived from ISTAT human demographic data, applying a pragmatic dog‐to‐human ratio to approximate provincial dog populations. This approximation does not remove denominator uncertainty; therefore, the resulting metric should be interpreted as a cumulative notification rate suitable for comparative surveillance and operational risk stratification, rather than as a direct measure of true transmission intensity.

In this evolving scenario, shifting from prevalence‐ to incidence‐based indicators is essential to improve territorial risk classification and guide proportionate prevention and control strategies. The aim of this study was to provide updated, province‐level incidence estimates of CanL in Italy for the 2022–2023 biennium, the most recent period for which nationwide diagnostic submissions were available with consistent coverage, so as to support risk‐based canine surveillance that is better aligned with public health surveillance needs, enabling targeted prevention and control strategies within a One Health framework.

## 2 Materials and Methods

### 2.1 Data Collection and Study Population

A retrospective observational study was conducted to estimate the incidence of canine leishmaniosis (CanL) across all 108 Italian provinces during the biennium 2022–2023. Provinces were defined according to the official territorial classification provided by Istituto Nazionale di Statistica (ISTAT) (2023). Data on newly diagnosed cases were obtained from diagnostic submissions provided by the national network of Istituti Zooprofilattici Sperimentali (IIZZSS), university diagnostic services, and private veterinary laboratories, and, when available, from official regional surveillance reports of the Ministry of Health and Local Health Authorities (ASL). Records were aggregated at provincial level based on the dog's reported province of residence; when residence was unavailable, the province of the submitting veterinary practice was used as proxy. Because data sources differ in reporting completeness and intensity, estimates were interpreted as surveillance‐based incident detections rather than exhaustive counts of all infections. Imported cases were excluded from the analysis based on information available in the diagnostic submission forms (origin and/or movement history). A CanL incident detection was operationally defined as a newly recorded IFAT seropositive submission for *Leishmania infantum* with a titre ≥ 1:40, used as a harmonised surveillance threshold across data providers (WHO n.d.). As noted in the WOAH Terrestrial Manual (2021), a titre of 1:40 is suggestive of exposure and may not necessarily indicate established infection; therefore, this operational definition captures incident serological detections for surveillance purposes rather than confirmed infection status (Paltrinieri et al. 2010). IFAT was selected because it is widely used in Italy and supports a consistent operational definition across heterogeneous diagnostic data providers. Confirmatory methods (e.g., PCR/qPCR on appropriate clinical specimens and/or parasitological confirmation) may improve diagnostic specificity and case characterisation; however, such results were not systematically available across all contributing laboratories and were therefore not incorporated into a harmonised national‐scale operational definition. Due to the absence of a standardised national canine registry, provincial dog populations were approximated using ISTAT (2023) human demographic data and an assumed dog‐to‐human ratio of 1:5.

### 2.2 Incidence and Data Analysis

The primary outcome was a biennial cumulative notification rate (hereafter, incidence proxy) calculated for each province as the number of newly diagnosed CanL cases detected through passive diagnostic submissions during 2022–2023 divided by the estimated canine population and expressed per 10,000 dogs. As individual dog‐time at risk and systematic active case finding were not available, this indicator should be interpreted as a surveillance‐based measure for comparative purposes rather than as a direct estimate of transmission intensity. Incidence values are presented as cumulative over the full biennium; an annualised estimate (biennial rate divided by two) is provided in Table S1. This standardised computation applies the same denominator construction across provinces; however, heterogeneity in testing intensity, access to diagnostics, and passive surveillance sensitivity likely remains and may contribute to geographic differences in detected cases. Incidence values were subsequently stratified into five predefined operational risk categories (minimal: 0–10; low: 11–40; moderate: 41–80; high: 81–160; very high: > 160 cases per 10,000 dogs). These cut‐offs were defined a priori as pragmatic thresholds for surveillance communication and prioritisation and should not be interpreted as biological transmission cut‐points. As an exploratory robustness check, provinces were also classified by incidence quintiles and compared with the predefined scheme (Table S3). Exact 95% confidence intervals (CIs) for provincial rates were calculated assuming Poisson‐distributed case counts and treating the estimated denominators as fixed. To assess robustness to denominator assumptions, sensitivity analyses were performed by recalculating provincial rates and risk classifications under alternative plausible dog‐to‐human ratios (1:4 and 1:6), alongside the baseline 1:5 scenario. In addition, a restricted analysis was conducted in regions covered by the Sistema Informativo Nazionale degli Animali da Compagnia (SINAC), part of the national Identification and Registration (I&R) system established under Legislative Decree No. 134 of 5 August 2022. The SINAC integrates regional companion animal registries into a national system aimed at ensuring identification, traceability, and support for veterinary public health activities. For these regions, the denominator was defined as the number of registered dogs in 2023, and incidence was calculated at regional level. The extent of risk‐category reclassification across scenarios is reported in Table S2.

Provinces were then classified according to these thresholds, and the distribution of risk classes across Italy was described in terms of absolute frequency.

Differences in the distribution of provinces across incidence risk categories were assessed according to geographical macro‐areas, defined as Northern Italy (Piemonte, Valle d'Aosta, Lombardia, Trentino‐Alto Adige, Veneto, Friuli‐Venezia Giulia, Liguria, Emilia‐Romagna), Central Italy (Toscana, Umbria, Marche, Lazio), and Southern Italy including Islands (Abruzzo, Molise, Campania, Puglia, Basilicata, Calabria, Sicilia, Sardegna). Statistical significance was evaluated using the chi‐square test of independence (*χ* ^2^), with *p* < 0.05 considered significant. Given the ordinal nature of incidence categories, a linear‐by‐linear association (trend) test was also computed as a sensitivity analysis.

Descriptive mapping and graphical visualisation were used to highlight spatial heterogeneity and support the territorial interpretation of surveillance‐based patterns. The cartographic representation of incidence classes was produced using QGIS software (version 3.24.1; QGIS Development Team, Open Source Geospatial Foundation Project).

## 3 Results

The incidence proxy of canine leishmaniosis (CanL) was estimated for all 108 Italian provinces for the 2022–2023 biennium. Provincial values ranged from 0 to nearly 500 cases per 10,000 dogs. Provinces were classified into predefined operational categories based on the incidence proxy. Extreme values were observed in a limited number of provinces and should be interpreted cautiously, as this incidence proxy may be influenced by estimated denominators and heterogeneous testing intensity. Based on calculated rates, 49 provinces (45.4%) were classified as minimal, 30 (27.8%) as low, 21 (19.4%) as moderate, 4 (3.7%) as high, and 4 (3.7%) as very high. Overall, almost three‐quarters of Italian provinces (79/108; 73.1%) fell within the minimal or low incidence categories, while 21 provinces (19.4%) were classified as moderate. Only 8 provinces (7.4%) exceeded 81 cases per 10,000 dogs, corresponding to the high (*n* = 4; Roma, Rieti, Agrigento, Palermo) and very high (*n* = 4; Imperia, Savona, Siracusa, Catania) operational strata. The geographical distribution of provinces across risk classes is reported in Table 1.

**TABLE 1:** Distribution of Italian provinces by canine leishmaniosis (CanL) risk class (2022–2023), with geographical breakdown.

Risk class	Incidence range (cases/10,000 dogs)	Number of provinces	Northern Italy	Central Italy	Southern Italy and Islands
Minimal	0–10	49 (45.4%)	31	10	8
Low	11–40	30 (27.8%)	15	10	5
Moderate	41–80	21 (19.4%)	2	8	11
High	81–160	4 (3.7%)	0	2 (Roma, Rieti)	2 (Agrigento, Palermo)
Very high	> 160	4 (3.7%)	2 (Imperia, Savona)	0	2 (Siracusa, Catania)
Total		108 (100%)	50	30	28

*Note:* Provinces (*n* = 108) were classified into five incidence risk classes according to newly diagnosed CanL cases per 10,000 dogs. Distribution is shown by macro‐area (North, Central, South and Islands).

Province‐specific incidence proxy estimates with exact 95% confidence intervals are reported in Table S1, and sensitivity analyses under alternative denominator assumptions are reported in Table S2. To assess the robustness of the incidence‐based stratification to denominator assumptions, we examined the stability of provincial stratum assignment across the three dog‐to‐human ratio scenarios (1:4, 1:5, and 1:6). Across the three scenarios, most provinces retained the same operational stratum, whereas a smaller subset was reclassified under at least one alternative denominator assumption; when reclassification occurred, it was limited to adjacent strata. These findings indicate that denominator assumptions influenced the exact placement of some provinces near category boundaries more than the identification of the principal higher‐burden areas. Comparison with the alternative quintile‐based stratification showed that the predefined operational scheme did not reproduce the empirical distribution of rates exactly, as expected from a distribution‐based approach, but did not materially alter the identification of the main higher‐incidence areas (Table S3). To address denominator uncertainty analytically in a higher‐data‐quality subset, we performed a restricted comparison in regions covered by the National Companion Animal Information System (SINAC), using registry‐based canine population denominators. This restricted comparison included 11 of the 20 Italian regions (55.0%) for which registry‐based canine population data were available through SINAC. When regional incidence was recalculated using registered canine populations as denominators, absolute incidence estimates were consistently higher than those obtained using the demographic proxy, owing to the smaller size of registry‐based denominators. However, despite these differences in absolute values, the relative spatial ordering remained highly stable. The correlation between proxy‐based and SINAC‐based regional incidence rankings was very strong (Spearman's ρ = 0.982; *p* < 0.001). Among the 11 SINAC‐covered regions, 7 retained exactly the same rank under both approaches, while the remaining 4 differed by only one position. Importantly, Liguria and Sicily remained the two highest‐ranking regions in both analyses. These findings indicate that, although absolute incidence estimates are sensitive to denominator specification, the proxy‐based framework is more robust for broad spatial prioritisation than for estimation of absolute incidence values. Detailed regional comparisons, corresponding risk‐class assignments, and ranking concordance are provided in Table S4. Descriptive external concordance with independent epidemiological, entomological, and ecological evidence is summarised in Table 2. Although formal external validation of absolute incidence estimates was not feasible, the higher‐incidence areas identified by the proxy‐based framework, particularly Sicily, Lazio, and Liguria, were descriptively concordant with previously reported evidence of established canine transmission and/or vector presence.

**TABLE 2:** Qualitative comparison between the incidence‐based classification proposed in this study and independent epidemiological, entomological, and ecological evidence of canine leishmaniosis in Italy.

Area	Incidence pattern identified in this study	Independent evidence supporting consistency	Key external support
Sicily (Agrigento, Palermo, Siracusa, Catania)	High‐ and very high‐incidence provinces	Consistent with a long‐established endemic focus, with high canine prevalence and sustained vector circulation	Maroli et al. 2008; Lisi et al. 2014; Verso et al. 2017; Bruno et al. 2022
Lazio (Roma, Rieti)	High‐incidence provinces	Consistent with persistent transmission in a historical endemic area, supported by canine exposure and competent vectors in both coastal and inland settings	Rombolà et al. 2021; Barlozzari et al. 2015; Maroli et al. 2008
Liguria (Imperia, Savona)	Very high‐incidence provinces	Compatible with stable endemicity in north‐western Italy and with previous evidence of canine foci and vector spread	Ferroglio et al. 2010; Maroli et al. 2008
Northern Italy	Mainly minimal/low incidence, with some moderate‐incidence provinces	Consistent with overall lower incidence, but with emerging or established focal transmission in some northern areas	Todeschini et al. 2024; Moirano et al. 2020; Gradoni et al. 2022
Italy overall	North–south gradient, with north‐western hotspots	Coherent with the established national pattern of higher endemicity in southern and insular areas and focal expansion in the north	Maroli et al. 2008; Moirano et al. 2020; Carvalho et al. 2024

## 4 Discussion

In Italy, the epidemiological monitoring of leishmaniasis differs substantially between canine and human populations. In dogs, which represent the primary domestic reservoir of *Leishmania infantum*, surveillance has traditionally relied on prevalence; that is, the proportion of seropositive animals within the tested population at a given time. However, prevalence estimates derived from diagnostic submissions are strongly influenced by sampling strategies, often enriched with clinically suspect animals, which may limit representativeness and comparability across settings. In contrast, incidence, defined as the number of new cases occurring in a defined population over a specified time frame, can provide temporally sensitive information on newly detected cases for surveillance purposes. It may support the detection of temporal changes in surveillance patterns and newly detected cases, as well as the evaluation of preventive and control strategies. As expected for a chronic infection with persistent serological positivity, prevalence and incidence are not directly comparable in magnitude and reflect different epidemiological constructs. This divergence is consistent with the chronic nature of CanL and does not, by itself, indicate bias in prevalence estimates. In the present study, incidence should be interpreted as a surveillance‐based proxy derived from passive case detection and an estimated denominator; its value lies in complementing prevalence with temporally sensitive information for stratification and comparative mapping. Human leishmaniasis surveillance, by contrast, is routinely based on incidence indicators, typically expressed as new cases per 100,000 inhabitants per year. However, the epidemiological meaning of incidence differs between hosts: in humans it largely reflects clinically apparent disease and healthcare‐based detection, whereas in dogs (reservoir host) incident detections reflect a mix of infection pressure, clinical presentation, veterinary access, and testing practices. This methodological discrepancy, while reflecting the different epidemiological roles of humans and dogs, limits the integrated assessment of risk and complicates predictions of transmission dynamics. The retrospective analysis by Bruno et al. (2022) illustrates this disparity, reporting a corrected canine prevalence of 34.4% against a cumulative human incidence of 1.04 cases per 100,000 inhabitants (Bruno et al. 2022). Such findings reinforce the need for harmonised One Health frameworks in which canine surveillance also relies on incidence‐based measures, ideally through active monitoring schemes involving sentinel cohorts or representative samples.

Comparable evidence is provided by Moirano et al. (2022), who analysed the spatio‐temporal pattern and meteo‐climatic determinants of incident cases of human visceral leishmaniasis in Italy (Moirano et al. 2022). In addition, a retrospective analysis from the Bologna Local Health Unit documented the re‐emergence of autochthonous human leishmaniasis in northern Italy over 2004–2022 (Todeschini et al. 2024), providing independent epidemiological context consistent with northward expansion dynamics. Together with the present results, their findings support the utility of incidence‐based indicators for capturing temporal changes in surveillance patterns and identifying areas where incident detections concentrate over time. Furthermore, at the European scale Carvalho et al. (2024), developed a climatic suitability indicator for *Leishmania infantum* transmission, demonstrating its strong association with both human incidence and canine seroprevalence, and revealing a northward expansion of suitable areas linked to climate change (Carvalho et al. 2024).

**FIGURE 1:**
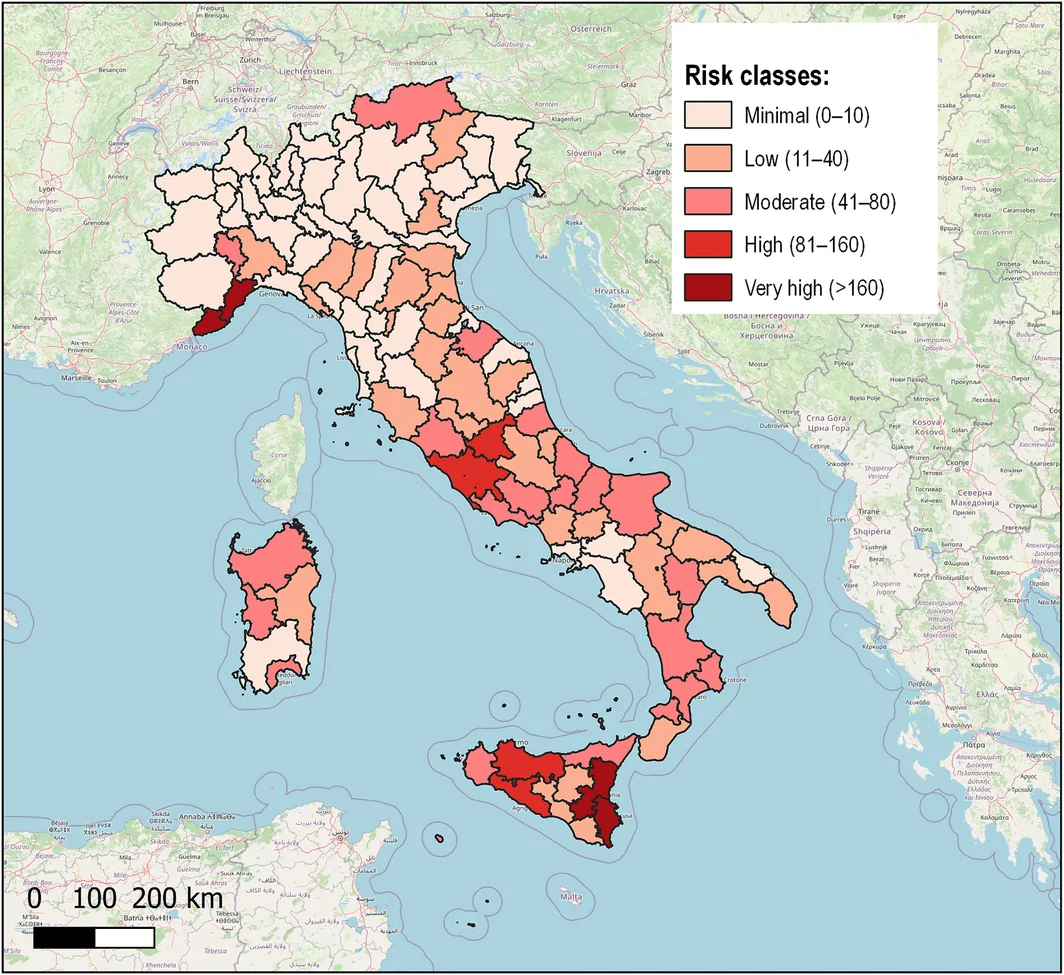
Provincial incidence of canine leishmaniosis (CanL) in Italy (2022–2023). Provinces are stratified into five epidemiological risk classes: Minimal (0–10), low (11–40), moderate (41–80), high (81–160), and very high (> 160) cases per 10,000 dogs.

The spatial distribution of CanL incidence across Italian provinces, as shown in Figure 1, highlights marked heterogeneity at the provincial level. Most northern provinces fell within the minimal and low operational incidence strata, whereas higher‐incidence strata were predominantly concentrated in Southern Italy and Sicily; however, localised hotspots were also identified in north‐western Italy, particularly in Liguria (Imperia and Savona). Importantly, this spatial pattern was broadly consistent with independent epidemiological, entomological, and ecological evidence reported for Italy, particularly for Sicily, Lazio, Liguria, and selected northern focal settings, as summarised in Table 2. This descriptive external concordance supports the epidemiological plausibility of the observed pattern, while not constituting formal validation of absolute incidence values. These spatial concentrations should nevertheless be interpreted cautiously, as provincial strata may reflect not only underlying transmission but also passive case detection, heterogeneous testing intensity, and uncertainty in estimated denominators. The map is therefore most informative for broad spatial prioritisation and identification of higher‐burden areas rather than for fine distinctions among neighbouring provinces located close to stratum boundaries. Human notifications reflect clinically apparent disease and healthcare‐based detection and cannot be used to directly validate canine incidence strata; formal cross‐validation would require integrated, spatially harmonised One Health datasets. Likewise, standardised province‐level indicators of vector presence or abundance were not available for quantitative validation within the scope of this study. The emergence of moderate‐risk provinces in central and northern Italy suggests ongoing changes in transmission dynamics, potentially linked to climate‐driven vector expansion (Dujardin et al. 2008; Maroli et al. 2008; Medlock et al. 2014) and increased canine mobility (Carla Maia and Cardoso 2015; Morosetti et al. 2020). This visual evidence supports the need for continuous monitoring and dynamic risk reclassification to capture evolving epidemiological patterns.

High and very high incidence categories were observed in eight provinces (Roma, Rieti, Agrigento, Palermo, Imperia, Savona, Siracusa, and Catania), representing 7.4% of the national total. These provinces share ecological and socio‐environmental features that may favour the persistence of *Leishmania infantum* transmission (Gradoni et al. 2017; Maroli et al. 2013). In Sicily, the high density of phlebotomine vectors and the combination of rural and peri‐urban habitats contribute to sustained endemicity, as confirmed by entomological and epidemiological surveys (Bruno et al. 2022). In Liguria, the provinces of Imperia and Savona exhibit climatic conditions characterised by mild winters and prolonged warm seasons, which are highly suitable for sand fly activity and have been associated with the northward spread of leishmaniasis (Alvaro et al. 2025; Morosetti et al. 2020; Rugna et al. 2017). The inclusion of Roma and Rieti among the high‐risk provinces highlights that central Italy is not exempt from intense transmission. For Roma, entomological studies have demonstrated stable sand fly populations with seasonal peaks that sustain parasite circulation (Bongiorno et al. 2003). For Rieti, both epidemiological and entomological data confirm that transmission is established despite the mountainous and relatively cool climate: serological surveys have reported canine prevalence up to 7.7% in some municipalities, human visceral leishmaniasis cases have been notified, and entomological investigations documented the presence of *Phlebotomus perniciosus* and other sand fly species in rural and peri‐urban areas (Barlozzari et al. 2015). These findings suggest that local microclimatic conditions and ecological niches can sustain vector populations even in areas traditionally considered marginal for sand fly colonisation. Conversely, in northern Italy most provinces fall into minimal or low categories, reflecting the relatively recent introduction of the parasite; nevertheless, ecological niche models and risk assessment studies have highlighted suitable habitats for sand fly establishment, suggesting the potential for future expansion (Morosetti et al. 2009). However, human notifications reflect clinically apparent disease and healthcare‐based detection and cannot be used to directly validate canine risk classes; formal cross‐validation would require integrated, spatially harmonised One Health datasets. Standardised province‐level indicators of vector presence/abundance were not available for quantitative validation within the scope of this study.

The stratification of provinces into five incidence‐based operational categories provides a practical framework for surveillance communication and prioritisation. The prevention and control measures summarised in Table 3 are guideline‐based and expert‐informed recommendations aligned with existing best practices; they are provided to support operational decision‐making within each risk stratum and were not empirically derived or calibrated from the incidence proxy thresholds used in the present analysis. The thresholds were defined a priori for operational risk communication under real‐world surveillance constraints and were not intended as biological transmission cut‐points. The overall spatial pattern, particularly the identification of the main higher‐incidence areas, was not materially altered by an alternative, distribution‐based stratification using incidence quintiles (Table S3).

**TABLE 3:** Classification of Italian provinces by CanL risk level based on estimated incidence (cases per 10,000 dogs) and corresponding recommended prevention and control measures.

Risk class	Incidence range (cases/10,000 dogs)	Recommended actions
Minimal	0–10	Passive surveillance; information campaigns for dog owners; repellents only for travel to higher risk zones; post‐exposure serological testing after return
Low	11–40	Seasonal repellent use; owner awareness campaigns; representative serological surveys; vaccination considered for outdoor dogs
Moderate	41–80	Full seasonal repellent coverage; vaccination for outdoor or at‐risk dogs; annual screening of donors; traceability of seropositive animals
High	81–160	Year‐round prophylaxis; systematic vaccination; semiannual screening of high‐risk dogs (breeders, donors, shelter animals); regional incidence mapping; continuous veterinary training
Very high	> 160	Continuous application of all preventive tools; mandatory screening for at‐risk animals; stray population control; integrated vector control (habitat management, adulticide treatments); active involvement of local authorities in One Health interventions

*Note:* Actions range from minimal surveillance and owner awareness in low‐incidence areas to the continuous and integrated application of all preventive strategies, including stray dog management and vector control, in very high‐risk settings.

The stratum‐based recommendations illustrate how prevention and control strategies can be progressively intensified according to the local surveillance context. In minimal‐ and low‐risk areas, the focus is primarily on passive or representative surveillance, complemented by owner awareness campaigns and seasonal repellent use, with the aim of detecting early parasite introductions while avoiding excessive reliance on preventive measures. In moderate‐risk provinces, the implementation of systematic vaccination in outdoor or at‐risk dogs, together with annual screening of donors, reflects the need to manage a consolidated endemic situation while ensuring the traceability of seropositive animals. High‐risk areas require a shift to year‐round prophylaxis, systematic vaccination, and semiannual screening of the most exposed categories, supported by regional incidence mapping and continuous veterinary training. Finally, in very high‐risk contexts, the integrated application of all available tools, including stray dog control and vector management, becomes indispensable, requiring the active involvement of local authorities within a One Health framework.

The stratification of recommendations is consistent with the prevention and control pathways described by Miró et al. (2008), who documented the effectiveness of interventions based on topical repellents, vaccination (Otranto and Dantas‐Torres 2013), and integrated vector management (Miró et al. 2008). The present framework expands these indications by embedding them into a harmonised incidence‐based surveillance model, capable of supporting prioritisation of preventive measures according to surveillance strata and fostering a comprehensive One Health approach.

Despite the availability of preventive tools, one of the main critical issues in the current CanL surveillance landscape remains the absence of a coordinated national One Health strategy. Surveillance and control are largely delegated to regional administrations, resulting in heterogeneous practices and unequal access to diagnostic and preventive resources. Regions without formal CanL surveillance plans often rely on sporadic case reporting, with less standardised diagnostics and a higher risk of underestimation. This heterogeneity further supports the need for a harmonised incidence‐based surveillance model capable of capturing real‐time dynamics and providing a robust evidence base for public health decision‐making. Several limitations should be considered. First, the incidence values presented here represent a surveillance‐based proxy derived from passive diagnostic submissions and therefore reflect not only underlying infection occurrence but also heterogeneity in veterinary access, clinician awareness, testing propensity, and laboratory pathways across provinces. Moreover, differential underreporting across provinces is plausible due to heterogeneity in diagnostic access and testing propensity. Without harmonised metadata on testing volume/coverage, we could not quantify or adjust for under‐ascertainment; future surveillance should collect simple denominators (e.g., tests per 10,000 dogs) and proxy indicators of diagnostic availability (e.g., laboratory density). Second, case definition relied on a single serological criterion (IFAT), which may entail some diagnostic misclassification due to imperfect sensitivity/specificity; however, inter‐laboratory variability in cut‐off titres and reading criteria was mitigated by external quality assurance, as participating laboratories underwent WOAH Reference Laboratory proficiency testing. While confirmatory PCR/qPCR can improve diagnostic specificity and support case characterisation, molecular results were not systematically available across data providers and could not be incorporated into a harmonised national‐scale definition, highlighting the need for standardised multi‐test algorithms in future One Health surveillance.

Third, provincial denominators were approximated using a uniform dog‐to‐human ratio because a standardised national canine registry was not available. As human‐to‐dog ratios are likely to vary across settings, denominator uncertainty may inflate rates in provinces where the true dog population is higher than assumed and attenuate them where it is lower. Sensitivity analyses using alternative plausible ratios (1:4 and 1:6) partially addressed this issue, although uncertainty could not be fully eliminated. Reclassification affected 6.5% (1:4) to 15.7% (1:6) of provinces and was always limited to a single adjacent stratum (Table S2), suggesting relative stability of the stratification under plausible denominator scenarios.

To further address this limitation while preserving the national scope of the analysis, we conducted a restricted comparison in regions covered by the Sistema Informativo Nazionale degli Animali da Compagnia (SINAC), using registry‐derived canine population data. In this higher‐data‐quality subset, incidence estimates were substantially higher when registry‐based denominators were used, as expected from the smaller denominator size; however, the overall spatial pattern remained broadly consistent, with the same regions identified among the highest‐incidence areas. These findings indicate that absolute incidence estimates are sensitive to denominator assumptions, whereas the relative ranking of regions appears more robust across alternative denominator specifications. Fourth, although confidence intervals were provided for case‐count variability, they do not capture all sources of uncertainty inherent to passive surveillance (e.g., differential testing intensity and diagnostic access). Finally, comparisons of category distributions across macro‐areas should be interpreted cautiously given the ordinal nature of the strata and the spatial structure of provinces, which may partially violate independence assumptions underlying chi‐square tests. Imported cases were excluded based on information available in the diagnostic submission forms (origin and/or movement history).

In summary, our findings support integrating incidence‐based surveillance indicators alongside prevalence measures within a One Health framework to enhance temporal sensitivity and risk communication, while enabling more standardised spatial comparisons under real‐world surveillance constraints. Operationalisation within a One Health framework would primarily rely on harmonised case definitions and standardised electronic data sharing between veterinary and public health systems, with linkage to entomological and climatic information to support periodic risk reporting. Overall, the combined use of proxy‐based national estimates, restricted analyses in registry‐covered regions, and descriptive external concordance provides a pragmatic surveillance framework for broad spatial prioritisation, while explicitly acknowledging substantial uncertainty in absolute incidence estimates.

## 5 Conclusions

An incidence‐based framework for canine leishmaniosis (CanL) in Italy provides a pragmatic, nationally scalable approach to describing the distribution of surveillance‐based incident detections.

By applying a standardised denominator construction based on ISTAT demographic data and routinely available diagnostic submissions, the analysis provides an operational, nationally comparable stratification framework to support region‐specific surveillance communication and prioritisation. The novelty of the approach lies primarily in its national‐scale operational implementation, rather than in conceptual innovation. The analysis reveals a heterogeneous spatial pattern with clustering and a north–south gradient. Higher categories were predominantly observed in Southern Italy and Sicily; however, very high provinces were also identified in North‐Western Italy (Liguria), notably Imperia and Savona, and moderate‐category provinces were observed across central and northern areas. These findings have implications for planning targeted interventions and for prioritising surveillance in areas where incident detections concentrate. Although this analysis was not designed to forecast future trajectories, the observed patterns are consistent with prior evidence that vector suitability and canine mobility may contribute to changing distributions over time. Incorporating an incidence proxy can strengthen the epidemiological basis for strategic planning by adding temporal sensitivity on newly detected cases, particularly in a context characterised by heterogeneous diagnostic capacities and uneven implementation of CanL surveillance across regions. Prevalence and incidence‐based indicators should be considered complementary: prevalence captures accumulated burden, whereas incidence proxies add temporally sensitive information on newly detected cases when interpreted within their operational constraints.

A unified, One Health‐based national surveillance strategy is therefore urgently needed. The current fragmentation, with parasite control delegated to regional administrations, creates inconsistencies in monitoring, reporting, and prevention. A centrally coordinated framework integrating human, animal, and environmental health could improve consistency in situational awareness and comparability across sectors and facilitate harmonised, timely public health responses. Ultimately, incidence‐based classification offers a useful complementary tool for guiding future surveillance efforts, promoting evidence‐informed decision‐making, and strengthening preparedness against this zoonosis.

## Funding

This work was supported by the Italian Ministry of Health through the following grants: IZS SI RC 06/23 and GR‐2019‐12369134.

## Ethics Statement

This study was based on the retrospective analysis of anonymized diagnostic data and publicly available demographic statistics from ISTAT. No experimental procedures were performed on animals, and no data allowing the identification of individual subjects were collected. In accordance with current Italian and European regulations, formal ethical approval was not required.

## Conflicts of Interest

The authors declare no conflicts of interest.

## Supporting information


**Table S1:** reports annual and biennial detected case counts, estimated canine population using a 1:5 dog‐to‐human ratio, and biennial incidence proxy per 10,000 dogs with 95% confidence intervals, together with annual‐equivalent rates.
**Table S2:** summarises sensitivity of risk‐category assignment to alternative denominator assumptions (1:4 and 1:6 dog‐to‐human ratios), listing provinces that change category and corresponding rates.
**Table S3:** reports agreement and reclassification metrics comparing fixed‐threshold risk classes with quintile‐based stratification.
**Table S4:** provides regional comparisons between proxy‐based incidence estimates and incidence recalculated using SINAC registered dog populations as denominators in SINAC‐covered regions, including corresponding proxy‐based and SINAC‐based risk‐class assignments, regional rankings under both approaches, and rank differences.

## Data Availability

The data that support the findings of this study are not publicly available due to legal and institutional restrictions (e.g., institutional agreements, privacy protection). Aggregated anonymised data and the analytical scripts used are available from the corresponding author (E.O.) upon reasonable request.
